# Can Experience Improve Hospital Management?

**DOI:** 10.1371/journal.pone.0106884

**Published:** 2014-09-24

**Authors:** Haruhisa Fukuda, Kazuhide Okuma, Yuichi Imanaka

**Affiliations:** 1 Department of Health Care Administration and Management, Graduate School of Medical Sciences, Kyushu University, Higashi-ku, Fukuoka, Japan; 2 Department of Healthcare Economics and Quality Management, School of Public Health, Graduate School of Medicine, Kyoto University, Yoshida Konoe-cho, Sakyo-ku, Kyoto, Japan; Azienda Ospedaliero-Universitaria Careggi, Italy

## Abstract

**Background:**

Experience curve effects were first observed in the industrial arena as demonstrations of the relationship between experience and efficiency. These relationships were largely determined by improvements in management efficiency and quality of care. In the health care industry, volume-outcome relationships have been established with respect to quality of care improvement, but little is known about the effects of experience on management efficiency. Here, we examine the relationship between experience and hospital management in Japanese hospitals.

**Methods:**

The study sample comprised individuals who had undergone surgery for unruptured abdominal aortic aneurysms and had been discharged from participant hospitals between April 1, 2006 and December 31, 2008. We analyzed the association between case volume (both at the hospital and surgeon level) and postoperative complications using multilevel logistic regression analysis. Multilevel log-linear regression analyses were performed to investigate the associations between case volume and length of stay (LOS) before and after surgery.

**Results:**

We analyzed 909 patients and 849 patients using the hospital-level and surgeon-level analytical models, respectively. The odds ratio of postoperative complication occurrence for an increase of one surgery annually was 0.981 (P<0.001) at the hospital level and 0.982 (P<0.001) at the surgeon level. The log-linear regression analyses showed that shorter postoperative LOS was significantly associated with high hospital-level case volume (coefficient for an increase of one surgery: −0.006, P = 0.009) and surgeon-level case volume (coefficient for an increase of one surgery: −0.011, P = 0.022). Although an increase of one surgery annually at the hospital level was statistically associated with a reduction of preoperative LOS by 1.1% (P = 0.006), there was no significant association detected between surgeon-level case volume and preoperative LOS (P = 0.504).

**Conclusion:**

Experience at the hospital level may contribute to the improvement of hospital management efficiency.

## Introduction

“Practice makes perfect” is a widely accepted axiom, but the specifics of this concept can vary among the different industries. In the 1960s, an analysis of the aircraft industry demonstrated that the labor input required to manufacture one aircraft was reduced by 20% whenever the manufacturing quantity was doubled [1], indicating the existence of “learning curve” effects in this field. A decade later, the Boston Consulting Group discovered that production and sales costs were also associated with manufacturer experience (represented by accumulated production quantity); this relationship between experience and efficiency was described as an “experience curve” [2]. Possible determinants of experience curve effects include (1) improvement in yield rates, (2) improvement in employee efficiency, (3) improvements in work specialization and methods of operation, (4) changes to the distribution of utilized resources, and (5) standardization of products and services.

Studies within the health care industry have demonstrated that higher patient case volumes are associated with better outcomes, such as shorter length of stay (LOS) [3]–[5] and lower hospital charges [3]. This suggests that experience curve effects may also apply to the field of health care provision, possibly through two separate effects: the first effect is where an increase in experience improves yield rates, given by Determinant 1 of the experience curve effects as outlined above. The second effect is where an increase in experience improves hospital management, given by Determinants 2 to 5. Several studies have provided evidence supporting the existence of experience curve effects in health care by documenting associations between increased hospital or physician experience and reductions in postoperative complications and mortality rates [6]–[12]. However, it remains unclear if the effects are due to improvements in yield rate or improvements to hospital management efficiency.

Therefore, this study attempts to first confirm the traditional hypothesis that “experience improves yield rates” in a health care setting and to verify a new hypothesis that “experience improves hospital management” through an analysis of hospital-level and surgeon-level surgical experience, represented by the respective case volumes at each level. If the first hypothesis holds true, increases in hospital-level and surgeon-level case volumes should be accompanied by reductions in postoperative LOS, partly due to improvements arising from increased yield rates (such as reductions in the occurrence of complications). If the second hypothesis holds true, increases in hospital-level case volumes should be accompanied by reductions in preoperative LOS due to improvements arising from increased hospital management efficiency. Since we assume that management efficiency (given by determinants 2–5) is associated with hospital-level experience rather than surgeon-level experience, preoperative LOS would be expected to be associated with hospital-level case volume, but not with surgeon-level case volume. In our study, preoperative LOS was used as a representative indicator of hospital management efficiency, based on the assumption that preoperative LOS should generally be kept to a minimum to reduce unnecessary costs and utilization of medical resources.

## Methods

### Data Sources

The database used in this study was constructed from two different data sources.

Hospitals in Japan that use the comprehensive payment system known as the Diagnosis Procedure Combination/Per-Diem Payment System (DPC/PDPS) are mandated to produce and submit a combination of claims and clinical data to the national government. These data are known as DPC data, and were used as one of the data sources for this study. DPC data include some information on medical treatment content, patient characteristics, and patient outcomes. The data were obtained from the Quality Indicator/Improvement Project (QIP), which is managed by the Department of Healthcare Economics and Quality Management, Kyoto University. The QIP database is constructed using hospitals that voluntarily participate in the program, and the data are produced in the same format as the data submitted to the government. Although the period for data submission to the government is from July to December each year, the QIP also collects data throughout the year from a portion of the institutions.

Because not all hospitals (including QIP hospitals) produce DPC data throughout the year, there are severe limitations in obtaining information regarding hospital-level and surgeon-level annual case volumes. Therefore, this study used a second data source in the form of a questionnaire survey targeting participant hospitals. Using this survey, we identified the surgeon at each participant hospital who performed the highest number of abdominal aortic aneurysm (AAA) surgeries in FY2008. We then determined the total number of AAA surgeries conducted by each of these surgeons during the 3-year period between April 1, 2006 and December 31, 2008, and used this number as the surgeon-level case volume of each hospital. We also calculated the total number of AAA surgeries performed by all surgeons at each participant hospital during the 3-year period, and used this number as the hospital-level case volume. Therefore, in hospitals with two or more surgeons that perform AAA surgeries, the surgeon-level case volume would be lower than the hospital-level case volume. If there is only one surgeon in a hospital, the surgeon-level case volume would be equal to the hospital-level case volume.

### Study Subjects

We focused on individuals who had undergone surgery for unruptured AAA and had been discharged from the participant hospitals between April 1, 2006 and December 31, 2008. Using the DPC data, unruptured AAA cases were identified as those with the disease name “unruptured abdominal aortic aneurysm” in any of the following disease designations: “principal disease”, “disease resulting in admission”, “disease that required the most medical resources during hospitalization”, “disease that required the second most medical resources during hospitalization”, or “comorbidity present on admission”; alternatively, AAA cases were identified if their records contained the International Classification of Diseases 10th revision (ICD-10) code “I714”. However, patients were excluded if they had died during hospitalization or presented with other conditions of the aorta (in addition to AAA). Furthermore, patients were identified as subjects if their records contained the surgery codes K5607 (Abdominal aorta surgery [reconstruction of branching blood vessels]) or K5608 (Abdominal aorta surgery [others]). Patients who had undergone other surgeries in addition to unruptured AAA surgery were excluded from analysis. Patients whose records indicated preoperative LOS durations of 0 days were also excluded because these values could not be logarithmically transformed, which was required for the statistical analysis described below.

### Variables

This study was conducted using the following three response variables: preoperative LOS (days), postoperative LOS (days), and postoperative complication occurrence. The two LOS variables were calculated from DPC data using the hospitalization periods before and after the date of unruptured AAA surgery. Postoperative complications were identified by first extracting all DPC data items that indicated post-admission complications in the study subjects, and then identifying the following complications as those associated with AAA surgery: cardiovascular diseases (blood pressure disorder, heart failure, ischemic heart disease, and arrhythmia), respiratory diseases (pneumonia, respiratory failure, pulmonary collapse, and pulmonary embolism), shock and diseases of the vital organs (multiple organ dysfunction syndrome or shock, bleeding diathesis, cerebrovascular disease, and postoperative nerve degeneration), gastrointestinal diseases (gastric ulcer, gastritis, or gastrointestinal hemorrhage; ileus; liver disease; pancreatitis; and iatrogenic harm due to thoracic surgery), urologic diseases (postoperative renal failure, urinary tract infection, neurogenic bladder dysfunction, and dysuria), and postoperative infections (surgical site infection, postoperative infection of unknown cause, and other surgical and wound complications).

The two exposure variables used were hospital-level case volume and surgeon-level case volume. Surgeon-level case volume was calculated as the case volume of the most experienced surgeon (highest annual case volume) at each hospital. The covariates to account for patient variations were obtained from DPC data and included patient sex, age at admission, emergency admission, and comorbidities present at admission. Comorbidities present at admission were analyzed using the Charlson comorbidity index [13], [14].

### Statistical Analysis

The three response variables were preoperative LOS (days), postoperative LOS (days), and postoperative complications; the exposure variables were hospital-level case volume and surgeon-level case volume. Six analytical models were developed based on the different combinations of these variables.

First, hospitals were categorized into the following three groups based on their annual case volumes: ≤19 cases, 20–29 cases, and ≥30 cases. Using ANOVA and Chi-squared tests, the associations between case volumes and the covariates were examined.

Next, we analyzed the association between case volume (both at the hospital and surgeon level) and postoperative complications using multilevel logistic regression analysis. Our database comprises hierarchical data composed of individual health care institutions, with patients nested within each institution. Because employee-associated factors, allocation of facilities, and treatment processes are likely to vary to a large degree among the hospitals, the associations between case volume and patient outcomes are also likely to be inconsistent at the hospital level. In order to account for these hospital-level variations, we employed a multilevel model for analysis, using case volume as the fixed effect and the individual hospitals as the random intercept.

Thirdly, the response variables of preoperative and postoperative LOS were logarithmically transformed before being analyzed using multilevel regression models to investigate their associations with case volume.

Statistical significance was set at P<0.05. Statistical analyses were performed using Stata version 13.1 (StataCorp, College Station, Texas, USA).

### Ethics Statement

This study was approved by the Ethics Committee of the Graduate School and Faculty of Medicine, Kyoto University. The study was conducted with a waiver of patient consent. Patient records/information was anonymized and de-identified prior to analysis. The study complied with the Ethical Guidelines for Epidemiological Research of the Japanese national government, which include guidelines on protecting patient anonymity, and all the necessary conditions were satisfied for informed consent to be waived.

## Results

### Response Rate

The questionnaire was sent to 107 QIP participant hospitals. Of these, 37 hospitals (response rate: 34.6%) responded regarding hospital-level case volume and 36 hospitals (33.6%) responded regarding patient-level case volume. After combining the results of the questionnaire responses with the DPC data, the numbers of study subjects included in the multilevel logistic regression models were 909 for hospital-level case volume (3 cases were excluded because their preoperative LOS was 0 days) and 849 for surgeon-level case volume (3 cases were excluded because their preoperative LOS was 0 days).

### Patient Characteristics

The basic characteristics of the study sample are shown in **Table 1**. The hospital-level case volumes ranged from 0.7 to 86.3 surgeries per year, with a mean of 34.0 surgeries per year. To describe the basic characteristics of the study subjects, patients were allocated into three groups based on their hospitals' case volumes. There were 273 cases from 22 hospitals (59.5% of all hospitals) with 19 or fewer surgeries per year, 257 cases from 8 hospitals with 20–29 surgeries per year, and 379 patients from 7 hospitals with 30 or more surgeries per year.

**Table 1:** Patient characteristics.

	Annual case volume by hospital			Total	P-value
	≤19 cases	20–29 cases	≥30 cases		
Number of patients	273	257	379	909	
Number of hospitals	22	8	7	37	
Female [N (%)]	54 (19.8%)	46 (17.9%)	69 (18.2%)	169 (18.6%)	0.830
Patient age,yr [Mean (SD)]	74.0 (7.7)	74.6 (7.9)	73.5 (8.4)	74.0 (8.1)	0.189
CCS at admission [Mean (SD)]	0.7 (0.8)	0.7 (1.0)	0.7 (0.9)	0.7 (0.9)	0.657
Emergency admission [N (%)]	26 (9.5%)	19 (7.4%)	26 (6.9%)	71 (7.8%)	0.438
Preoperative LOS, Days [Mean (SD)]	7.7 (7.7)	6.3 (7.0)	3.7 (2.7)	5.6 (6.1)	<0.001
Postoperative LOS Days [Mean (SD)]	21.6 (14.9)	19.0 (20.3)	17.9 (12.3)	19.3 (15.8)	0.012
Total LOS, Days [Mean (SD)]	29.3 (17.8)	25.3 (22.3)	21.6 (13.0)	25.0 (17.8)	<0.001
Postoperative complication ≥1 [N (%)]	171 (62.6%)	144 (56%)	152 (40.1%)	467 (51.4%)	<0.001

CCS, Charlson Comorbidity Score; LOS, length of stay.

The results showed no significant differences among the three groups with respect to patient sex, average age on admission, average Charlson comorbidity score on admission, and proportion of emergency admissions. However, hospitals with a high case volume tended to have a lower, albeit non-significant, proportion of emergency admissions. In contrast, higher case volume was found to be significantly associated with shorter postoperative LOS (P<0.001), shorter preoperative LOS (P = 0.012), and shorter total LOS (P<0.001).

### Multilevel Analysis: Case Volume and Complications

**Table 2** shows the results of the multilevel logistic regression analysis of the association between case volume and postoperative complications. An increase of one surgery annually at the hospital level was significantly associated with a reduction in postoperative complication occurrence (odds ratio [OR]: 0.981; P<0.001). The OR of postoperative complication occurrence for an increase of one surgery annually at the surgeon level was 0.982. Similar to the hospital-level analysis, this increase in case volume was significantly associated (P<0.001) with a reduction in postoperative complication occurrence. No significant association between emergency admission and postoperative complication occurrence was observed.

**Table 2:** Results of multilevel logistic regression analysis of the impact of case volume on postoperative complication occurrence.

	Outcome: postoperative complication					
	Hospital-level case volume (37 hospitals, 909 cases)			Surgeon-level case volume (36 hospitals, 849 cases)		
	Odds Ratio	95% CI	P-value	Odds Ratio	95% CI	P-value
Hospital-level case volume	0.981	0.975, 0.988	<0.001	-	-	-
Surgeon-level case volume	-	-	-	0.982	0.968, 0.997	0.016
Emergency admission	0.928	0.560, 1.538	0.771	0.911	0.549, 1.514	0.720
Charlson Comorbidity Score	1.497	1.284, 1.746	<0.001	1.527	1.304, 1.789	<0.001
Age	1.017	1.000, 1.034	0.051	1.017	1.000, 1.034	0.053

### Multilevel Analysis: Case Volume and Length of Stay

**Figure 1:**
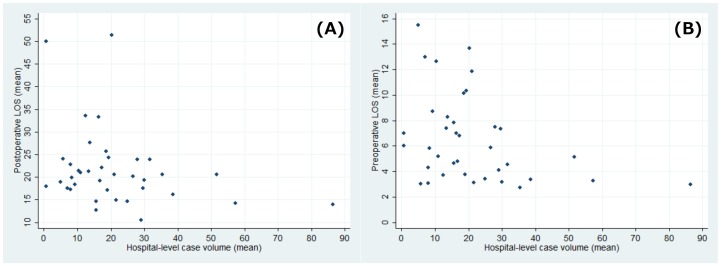
Scatter plots showing the mean annual hospital-level case volume against the mean postoperative length of stay (A) and the mean preoperative length of stay (B).

Scatter plots of hospital-level case volume against postoperative LOS and preoperative LOS (**Figures 1A and 1B**, respectively) illustrate negative relationships between case volume and both types of LOS. The scatter plots show wide variations in the data at lower case volumes, followed by a plateau in the effect of case volume on LOS; this plateau appeared between 35 and 50 operations per year. The results of the multilevel regression analysis estimating the effects of hospital-level or surgeon-level case volume on postoperative LOS are shown in **Table 3**. Because sex did not show any statistical association with LOS, it was excluded from the multivariable analysis. The regression analysis showed that shorter postoperative LOS was significantly associated with high hospital-level case volume (P = 0.009) and surgeon-level case volume (P = 0.022). An increase of one surgery annually at the hospital level was estimated to reduce postoperative LOS by 0.6%.

**Table 3:** Results of multilevel regression analysis of the impact of case volume on length of stay before and after AAA surgery.

	Outcome: Postoperative LOS						Outcome: Preoperative LOS					
	Hospital-level case volume (37 hospitals, 909 cases)			Surgeon-level case volume (36 hospitals, 849 cases)			Hospital-level case volume (37 hospitals, 906 cases)			Surgeon-level case volume (36 hospitals, 846 cases)		
	Coef.	95% CI	P-value	Coef.	95% CI	P-value	Coef.	95% CI	P-value	Coef.	95% CI	P-value
Hospital-level case volume	−0.006	−0.010, −0.001	0.009	-	-	-	−0.011	−0.020, −0.003	0.006	-	-	-
Surgeon-level case volume	-	-	-	−0.011	−0.020, −0.001	0.022	-	-	-	−0.006	−0.025, 0.012	0.504
Emergency admission	0.220	0.118, 0.321	<0.001	0.199	0.099, 0.298	<0.001	0.785	0.649, 0.921	<0.001	0.833	0.695, 0.971	<0.001
Charlson Comorbidity Score	0.057	0.027, 0.086	<0.001	0.062	0.033, 0.092	<0.001	0.018	−0.021, 0.056	0.374	0.028	−0.012, 0.068	0.168
Age	0.007	0.004, 0.01	<0.001	0.007	0.003, 0.010	<0.001	0.007	0.003, 0.012	0.002	0.008	0.003, 0.012	0.001

LOS, length of stay.

The associations between case volume and preoperative LOS are also shown in **Table 3**. The analysis showed that hospitals with high case volumes tended to have significantly shorter preoperative LOS (P = 0.006). An increase of one surgery annually at the hospital level was estimated to reduce preoperative LOS by 1.1%. In contrast, there was no significant association detected between surgeon-level case volume and preoperative LOS.

## Discussion

This study showed that increases in both hospital-level and surgeon-level case volumes were associated with reductions in postoperative LOS durations. The findings suggest that one of the reasons for the shorter postoperative LOS is the reduction of postoperative complications due to increased yield rates. In addition, the study showed that a reduction in preoperative LOS was associated with higher case volumes at the hospital level, but not at the surgeon level. An interpretation of these findings is that for unruptured AAA surgeries, the accumulation of experience may improve the technical skills of the surgeons and surgery teams. Furthermore, the results showed that only hospital-level case volume was significantly associated with reduced preoperative LOS, suggesting that experience at the hospital level may contribute to the improvement of hospital management.

The associations between case volume and patient outcomes have been addressed in previous studies, which have focused on procedures such as carotid endarterectomy [7], tumor resection [7], [8], coronary artery bypass grafting (CABG) [1], and AAA surgery [7], [9]–[12]. In this study, we opted to focus on unruptured AAA surgery cases for two reasons. The first reason is that it was possible to analyze the disease severity of AAA patients; the most important factor that can influence the severity of these patients is whether an aneurysm has ruptured or not. As our database uses ICD-10 classifications, we were able to distinguish unruptured AAA cases from those with ruptured AAA. In contrast, inconsistent availability of disease classification information for tumor resection cases in the DPC data renders it difficult to appropriately adjust for disease severity. Furthermore, claims data for CABG cases do not include information on the number and site of infarctions of each patient, which can greatly affect patient outcomes. These procedures are therefore unsuitable for analyses conducted using only claims data. The second reason for the selection of unruptured AAA cases as the study sample is that there is a high risk that patient conditions could quickly deteriorate without prompt treatment, compounded by the relatively high number of cases. Hospitals must therefore strive to perform AAA surgery as quickly as possible. In this way, consistently short preoperative LOS durations can indicate that a hospital is systematically able to conduct surgeries quickly after admission. For these reasons, unruptured AAA cases are suitable subjects for an investigation of the association between preoperative LOS (as a representative indicator of hospital management efficiency) and case volume.

To the best of our knowledge, there are currently 4 studies that have analyzed LOS as an outcome measure in AAA patients [3]–[5], [15]. However, all these studies have focused on total LOS durations, and none have addressed preoperative and postoperative LOS durations separately. Dardik et al. employed a univariable analysis on AAA patients, and reported that high surgeon-level case volumes were associated with reductions in LOS and health care expenditure [3]. Pronovost et al. reported that there was no significant association detected between hospital-level case volume and LOS [5]. Dimick et al. conducted a univariable analysis that showed hospitals with annual case volumes of 31 or more had a LOS that was one day shorter than hospitals with lower annual case volumes (P = 0.002) [4]. Wen et al. reported that an increase of one surgery in annual case volume would reduce LOS by 0.029 days for ruptured AAA and 0.012 days for unruptured AAA [5]. In contrast, our analysis showed that an increase of one surgery in annual case volume had an estimated reducing effect on postoperative LOS of 0.6%, indicating a reduction of 0.116 days. This large reducing effect observed in our study may be due to the comparatively long total LOS durations of our study sample (mean: 25.0 days), which was much longer than the total LOS durations reported in Wen et al. [5].

This study has several limitations that should be noted. The first is that although the study design attempted to account for disease severity (by adjusting for age, sex, emergency admissions, and comorbidity score); it is possible that the adjustment is inadequate. Previous studies have included the following variables for adjustments: age [3]–[5], [15]–[32], comorbidities [4], [5], [15]–[27], sex [4], [5], [15]–[19], [28]–[31], [33], medical specialty [16], [17], [27]–[29], certification [15], [28], [30], patient income [16], [17], intestinal ischemia [18], [26], type of hospital [15], [34], ASA class [32], location of hospital [34], and timing of surgery [16]. However, the three factors that consistently showed associations with the volume-outcome relationship in these previous studies are patient age, sex, and comorbidities. The inclusion of these factors in our analysis may therefore indicate that adjustments for disease severity may be sufficient. The second limitation is that due to the recent public release of hospital case volume information, it is possible that patients with relatively low disease severity (and therefore higher mobility) would congregate to hospitals with high case volumes due to perceived expertise and experience in providing treatment for specific diseases. As a result, hospitals with high case volumes may appear to have more favorable performances. In reality, these hospitals also had higher proportions of emergency admissions, but this relationship was not statistically significant. Despite our attempt to adjust for disease severity, reimbursement claims data do not include clinical information such as the size of the aneurysm or its detailed location. As we were unable to account for these factors, we could not determine if some hospitals tended to accept patients with lower severity. As a result, it is possible that there may be some degree of bias in the study due to data constraints. The third limitation is that a relatively large number of hospitals did not respond to the questionnaire survey (69 hospitals). As a result, there is a possibility that the data used in the analysis are not representative of all hospitals in the QIP. For example, the mean number of annual AAA surgeries per hospital in our sample was 8.2 cases. According to an annual survey conducted by the Japanese Association for Thoracic Surgery (JATS) in 2009 on all 582 hospitals in Japan accredited by the Japanese Board of Cardiovascular Surgery (JBCS), the mean number of type A acute aortic dissections conducted annually in 485 respondent hospitals (response rate 83.3%) was 7.4 cases [35]. The case volume of our study hospitals was therefore slightly higher than that of JBCS-accredited hospitals. Finally, this study focused on only one surgery type: AAAs. Although the inclusion of multiple surgery types may be useful for analysis, doing so would require highly complicated risk adjustments to account for the various disease severities accompanying each different surgery type. In addition, there would be practical limitations in acquiring the necessary data and examining the large patient populations required for multiple surgery analysis. For these reasons, we have focused on AAA surgery alone. These limitations should be considered when interpreting the findings of this study.

Despite some limitations, this study was able to provide new insight on the volume-outcome relationship, as we investigate whether experience can improve the effectiveness of hospital management through an analysis of preoperative and postoperative LOS. In the US, the functional differentiation of health care has advanced greatly, resulting in a substantial reduction in LOS durations in acute care hospitals. In that context, it would be difficult to conduct research that focuses on preoperative LOS as a means of evaluating hospital management. As this study was conducted in Japan, which has markedly protracted LOS durations when compared to the US and Europe, we were able to use this distinctive characteristic in order to elucidate the association between experience and hospital management.
